# The Role of Socioeconomic Status, Family Resilience, and Social Support in Predicting Psychological Resilience Among Chinese Maintenance Hemodialysis Patients

**DOI:** 10.3389/fpsyt.2021.723344

**Published:** 2021-09-30

**Authors:** Yuan Qiu, Yingying Huang, Yuxin Wang, Liya Ren, Hao Jiang, Liping Zhang, Chaoqun Dong

**Affiliations:** ^1^School of Nursing, Wenzhou Medical University, Wenzhou, China; ^2^The Second Affiliated Hospital of Wenzhou Medical University, Wenzhou, China

**Keywords:** maintenance hemodialysis, social support, family resilience, psychological resilience, socioeconomic status

## Abstract

**Objectives:** Evidence regarding the possible influence of social factors on psychological resilience among maintenance hemodialysis patients is scarce. The aim of this study was to explore the relationship among socioeconomic status, family resilience, and social support, and psychological resilience among Chinese maintenance hemodialysis patients.

**Methods:** This cross-sectional study was conducted in the hemodialysis centers of three comprehensive hospitals in China from September to December 2020 using convenience sampling. Two hundred fifty-eight patients receiving maintenance hemodialysis were investigated using a sociodemographic questionnaire, the Chinese version of the Medical Outcomes Study-Social Support Survey (MOS-SSS), Chinese Family Resilience Assessment Scale (C-FRAS), and Chinese version of the Conner and Davidson resilience scale (CD-RISC).

**Results:** Maintenance hemodialysis patients reported a low level of physical resilience, with a score of (58.92 ± 15.27). Hierarchical linear regression analysis showed that education level (β = 0.127, *p* = 0.018), maintenance of a positive outlook by the family (β = 0.269, *p* = 0.001), positive social interaction support from the family (β = 0.233, *p* = 0.002), and tangible support (β = −0.135, *p* = 0.037) were significantly associated with psychological resilience.

**Conclusion:** SES, family resilience and social support may be potential predictive factors of psychological resilience. Interventions to improve the family resilience and social support may be beneficial to promote the psychological resilience of Chinese maintenance hemodialysis patients.

## Introduction

Hemodialysis is the main form of renal replacement therapy in the terminal stage of chronic renal failure (1). According to the latest census data released in the China Kidney Disease Network Data Report (2), the prevalence of maintenance hemodialysis among patients with chronic renal failure in China is 402.18 per million. Hemodialysis treatment preserves the lives of patients with terminal stage renal failure but does not prevent the emotional suffering associated with chronic stress related to the disease burden, dialysis treatment, functional limitation, and fear of death (3–7). The incidence of emotional distress is higher in patients who received maintenance hemodialysis than in those with chronic kidney disease alone (8). It has been proposed that an individual's internal resources and external support play important roles in overcoming emotional distress during the treatment (9, 10).

Psychological resilience, defined as an individual's ability to actively mobilize all favorable factors to maintain or restore relatively stable mental and physical functions in the face of stressful life events and adversity (11), is widely recognized as an individual's competency and strength to successfully cope with stress (12). Higher psychological resilience is associated with greater acceptance of the disease, higher compliance with therapeutic regimens, and more favorable outcomes in patients with chronic renal diseases (13, 14). Lower psychological resilience is associated with emotional dysregulation (15) and variations in sensory processing (16, 17), which can increase the risk of suicidality in some patient populations (18). As cognitive flexibility is reported to be a critical factor to prevent negative outcomes and suicidal behavior in response to stressful life events (19), it is important to explore the psychological resilience of maintenance hemodialysis patients.

The systematic self-reflection model of resilience highlights the resilience resource of an individual, such as socioeconomic status (SES), family resources, and social support, which is one of the fundamental capacities for psychological resilience. The role of SES in the development of psychological resilience is contradictive. Wister et al. (20) suggests that individuals with higher SES have greater resilience, as they have greater social and economic resources available to them compared to individuals of lower SES. Other theorists (21) hypothesized that individual with low SES will exhibit prolonged, high-effort coping behavior to deal with emotional stress. To our best knowledge, there is no empirical study exploring the relationship of SES and psychological resilience among maintenance hemodialysis patients.

Social support, a multidimensional concept, is defined as the provision of psychological and material resources by caregivers, medical staff, and other social networks to benefit an individual's ability to cope with stress (22). The subjective perception of social support has been identified as a protective factor for psychological resilience in other populations, such as adolescents (23, 24), cancer patients (25, 26), and older individuals (27). However, the role of objective social support on psychological resilience is less clear. Objective social support can come in varying forms, including tangible support, informational/emotional support, positive social interaction support, and affectionate support (28). To better understand the mechanisms underlying the effects of social support, it is vital to understand the types of social support that are beneficial for psychological resilience.

Family resilience, one of the most important family resources, is defined as a family's ability to withstand and rebound from adversity and to become stronger and more resourceful (29). Previous studies have identified multiple dimensions of family resilience, such as family cohesion, family communication, a family's ability to make meaning of adversity, maintaining a positive outlook, utilizing social and economic resources, etc. (30). However, there are few studies exploring the effect of each aspect of family resilience on individual resilience. Only one recent study of Japanese hemodialysis patients found family communication was associated with higher psychological resilience, while family cohesion was not associated with individual resilience (31).

Some scholars caution that the protective or risk characteristic of psychological resilience depends on the context and meaning of each element, particularly how each factor is perceived by an individual (32). Therefore, the purpose of this study is to examine the predictive roles of SES, family resilience, and social support for psychological resilience among Chinese Maintenance hemodialysis patients. We hypothesized that socioeconomic status, family resilience, and social support will be positively associated with psychological resilience after controlling for demographic and clinical variables.

## Methods

### Participants

Two hundred eighty patients were recruited using the convenience sampling method in this study. The inclusion criteria were as follows: (1) age 18 years or above; (2) receiving hemodialysis regularly for more than 3 months; (3) no communication barriers; (4) willing to participate in this study. The exclusion criteria were: (1) physician-diagnosed psychiatric or mental disorders based on the Diagnostic and Statistical Manual of Mental Disorders (DSM-IV, TR) (33), such as schizophrenia, bipolar disorder, and depression; (2) neurological disorders or cognitive impairments (e.g. delirium, dementia); and (3) inability to communicate verbally or complete the questionnaires. The investigator carefully collected the participants' psychiatric histories by reviewing their medical records and questioning the patients or their family members. Of the 280 eligible maintenance hemodialysis patients invited to participate, 22 eligible participants declined to participate due to a lack of interest or fatigue. The remaining 258 participants all returned complete and valid questionnaires, resulting in a valid sample size of 258 (participation rate = 92.14%).

### Procedure

After obtaining ethical approval for the study from the Affiliated Hospital of the Medical University Ethics Committee (No. 2020198), this cross-sectional study was conducted in accordance with the Declaration of Helsinki in the hemodialysis center of three comprehensive hospitals in Zhejiang Province, China, from September to December 2020. Data were collected using structured questionnaires with the cooperation of hemodialysis center nurses. A trained investigator identified potential eligible patients by reviewing their medical records and asking about their psychiatric history for initial screening. The eligible participants were informed of the purpose and procedure of the study. Written informed consent was obtained from all participants before starting any procedures, and the confidentiality of their information was guaranteed. The participants were instructed to complete the pen-and-paper self-reported questionnaires in a quiet room before the hemodialysis treatment. For participants who could not write, the investigator read out the questionnaire items verbatim without adding further explanation and completed the questionnaires according to the patient's responses. The entire survey process lasted for 20–30 min, and the investigator immediately reviewed the questionnaires and asked the patients to provide any missing items after each survey was completed. All participants were provided with a small gift of a cookie valued at $1 as compensation.

### Measures

#### Demographic and Clinical Variables

The following variables were assessed: age, gender, employment status, marital status, medical insurance (yes/no), disease duration, dialysis duration, and frequency of hemodialysis and comorbidities.

#### SES

Two indicators for SES were assessed. Financial status was measured as the monthly household income per capita and was coded into four categories, from 1 (<2,000 RMB) to 4 (>6,000 RMB). Education was measured as the highest grade of schooling completed and was coded into four categories, from 1 (primary school) to 4 (college or higher).

#### Social Support

The Chinese version of the Medical Outcomes Study–Social Support Survey (MOS-SSS) (34), a 19-item scale, was used to assess the extent to which each individual had the support of others to cope with their stressful situation during the course of chronic disease. MOS-SSS recognizes the following four types of social support: informational/emotional support (eight items, expression of positive effect and empathetic understanding/offering of advice, information, guidance or feedback), tangible support (four items, provision of material aid or behavioral assistance), positive social interactive support (four items, the availability of other persons to entertain the patient), and affectionate support (three items, expressions of love and affection). Participants are asked to indicate how often each type of social support is available to them when they need it. A 5-point response ranging from 1 = none of the time to 5 = all of the time is used. The total score ranges from 19 to 95, with higher scores indicating higher levels of social support. The Cronbach's α coefficient was 0.944 for the total score in the present study.

#### Family Resilience

Family resilience was measured using the 44-item Chinese version of the Family Resilience Assessment Scale (C-FRAS) (30), which comprises four subscales: family communication and problem solving (27 items), utilizing social and economic resources (eight items), maintaining a positive outlook (six items), and ability to make meaning of adversity (three items). Each item is rated on a four-point Likert scale from 1 = strongly disagree to 4 = strongly agree, with total scores ranging from 44 to 176. Higher scores indicate higher levels of family resilience. The Cronbach's α coefficient was 0.968 for the total score in the present study.

#### Psychological Resilience

Psychological resilience was assessed using the Chinese version of the Conner and Davidson resilience scale (CD-RISC) (35). The 25-item CD-RISC contains three subscales, namely tenacity (13 items), strength (eight items), and optimism (four items). It uses a Likert five-point scale from 0 = not true at all to 4 = true all the time, with a total score of 0–100. Higher scores indicate higher levels of psychological resilience. The Cronbach's α coefficient of the scale in the present study was 0.927.

### Statistical Analysis

All statistical analysis was performed using IBM SPSS 25.0 (IBM Corp., Armonk, NY, USA). The *t*-test or one-way ANOVA was used to compare the groups. Pearson's *r* correlations were calculated to test for unadjusted associations between SES, social support, family resilience, and psychological resilience.

As the total scores of psychological resilience approached normality (*W* = 0.995, *p* = 0.479), hierarchical linear regression analyses were conducted with the sociodemographic and clinical variables in Step 1. SES was added in Step 2, family resilience was included in Step 3, and social support was entered in Step 4. Statistical significance was set at the level of 0.05 or less (two-tailed). Statistical significance was interpreted as a *p* < 0.05 (two-tailed).

## Results

### Descriptive Statistics

Two hundred fifty-eight maintenance hemodialysis patients (174 men and 84 women), with a mean age of 57.6 ± 13.83 years, submitted complete questionnaires. Participants were predominantly unemployed (*n* = 213, 82.6%), married/cohabitating (*n* = 228, 88.4%), with medical insurance (*n* = 253, 98.1%), and diagnosed with chronic renal failure for no <10 years (*n* = 137, 53.1%). Regarding the duration of hemodialysis, 48 (18.6%) participants had been treated for <1 year, 109 (42.2%) for 1–5 years (not including 5 years), 65 (25.2%) for 5–10 years (not including 10 years), and 36 (14%) for no <10 years. The mean duration of maintenance hemodialysis treatment was 58.38 months (SD = 48.33, range 4–236).

Regarding the SES, the sample comprised participants with education levels of primary school or below (*n* = 70, 27.1%), middle school (*n* = 97, 37.6%), high school (*n* = 59, 22.9%), and college or higher (*n* = 32, 12.4%). For financial status, 38 (14.7%) participants reported a family monthly income of <2,000 RMB, 93 (36.0%) reported a family monthly income of 2,000–4,000 RMB, 76 (29.5%) reported a family monthly income of 4,001–6,000 RMB, and 51 (19.8%) reported a family monthly income of >6,000 RMB. The characteristics of the study population are summarized in Table 1.

**Table 1:** Psychological resilience in relation to demographic, clinical, and SES characteristics (*N* = 258).

**Variable**	***N* (%)**	**Psychological resilience (Mean ± SD)**	***t*/*F***	***p***
**Demographic and clinical variables**				
Age			2.027	0.044
<60 years	130 (50.4%)	60.82 ± 15.54		
≥60 years	128 (49.6%)	56.99 ± 14.80		
Gender			1.355	0.177
Male	174 (67.4%)	59.82 ± 15.27		
Female	84 (32.6%)	57.07 ± 15.18		
Employment status			3.890	<0.001
Employed	45 (17.4%)	66.76 ± 13.99		
Unemployed	213 (82.6%)	57.27 ± 15.04		
Marital status			−2.122	0.035
Single/divorced/widow/separated	30 (11.6%)	53.40 ± 12.44		
Married/cohabitating	228 (88.4%)	59.65 ± 15.48		
***Medical insurance***				
Yes	253 (98.1%)	59.12 ± 15.25	−1.471	0.143
No	5 (1.9%)	49.00 ± 14.09		
Duration of disease			0.442	0.723
<1 year	13 (5.0%)	58.46 ± 16.78		
1– <5 years	53 (20.5%)	60.98 ± 15.28		
5– <10 years	55 (21.3%)	57.78 ± 16.23		
≥10 years	137 (53.1%)	58.63 ± 14.82		
Duration of hemodialysis			1.930	0.125
<1 year	48 (18.6%)	58.48 ± 16.03		
1– <5 years	109 (42.2%)	61.44 ± 15.46		
5– <10 years	65 (25.2%)	56.37 ± 15.15		
≥10 years	36 (14.0%)	56.50 ± 13.08		
Comorbidities			0.377	0.686
No	77 (29.8%)	58.22 ± 17.00		
One	119 (46.1%)	59.82 ± 13.85		
Two or more	62 (24.0%)	58.08 ± 15.76		
**SES variables**				
Education level			11.379	<0.001
Primary School and below	70 (27.1%)	52.29 ± 13.54		
Middle School	97 (37.6%)	58.01 ± 14.73		
High School/secondary school	59 (22.9%)	63.15 ± 13.40		
College or higher	32 (12.4%)	68.41 ± 16.97		
Monthly household income per capita			9.667	<0.001
<2,000 RMB	38 (14.7%)	51.74 ± 14.01		
2,000–4,000 RMB	93 (36.0%)	57.75 ± 14.78		
4,001–6,000 RMB	76 (29.5%)	58.01 ± 13.95		
>6,000 RMB	51 (19.8%)	67.76 ± 15.37		

### Association Between Demographic and Clinical Variables, SES, and Psychological Resilience

A significant difference in psychological resilience was found according to different demographic variables including age (*t* = 2.027, *p* = 0.044), occupational status (*t* = 3.890, *p* < 0.001), and marital status (*t* = −2.122, *p* = 0.035). A significant difference was also observed in the psychological resilience of patients with different educational levels (*F* = 11.379, *p* < 0.001) and different monthly household incomes per capita (*F* = 9.667, *p* < 0.001; see Table 1).

### Correlation Between Social Support, Family Resilience, and Psychological Resilience

The results of the correlation analysis are shown in Table 2. Significant correlations were observed between all four domains of social support and psychological resilience (*r* = 0.207–0.543, *p* < 0.01). The family resilience subscales also positively correlated with psychological resilience (*r* = 0.390–0.575, *p* < 0.01).

**Table 2:** Correlation analysis between social support, family resilience, and psychological resilience (*N* = 258).

	**Mean ± SD**	**1**	**2**	**3**	**4**	**5**	**6**	**7**	**8**	**9**	**10**	**11**
1. Tangible support	16.74 ± 3.40											
2. Informational/emotional support	24.85 ± 6.05	0.554^**^										
3. Positive social interaction support	12.71 ± 3.37	0.465^**^	0.767^**^									
4. Affectionate support	11.26 ± 2.40	0.688^**^	0.749^**^	0.644^**^								
5. FCPS	81.79 ± 7.85	0.308^**^	0.496^**^	0.443^**^	0.459^**^							
6. USER	21.89 ± 2.58	0.165^**^	0.343^**^	0.316^**^	0.216^**^	0.630^**^						
7. MPO	17.74 ± 2.12	0.276^**^	0.467^**^	0.439^**^	0.425^**^	0.825^**^	0.560^**^					
8. AMMA	9.03 ± 0.89	0.308^**^	0.432^**^	0.393^**^	0.370^**^	0.790^**^	0.565^**^	0.609^**^				
9. Tenacity	29.61 ± 8.41	0.141^*^	0.450^**^	0.484^**^	0.359^**^	0.531^**^	0.373^**^	0.545^**^	0.400^**^			
10. Strength	20.42 ± 5.23	0.209^**^	0.484^**^	0.540^**^	0.403^**^	0.519^**^	0.354^**^	0.557^**^	0.373^**^	0.892^**^		
11. Optimism	8.89 ± 2.67	0.331^**^	0.539^**^	0.526^**^	0.455^**^	0.480^**^	0.362^**^	0.482^**^	0.439^**^	0.677^**^	0.682^**^	
12. Psychological resilience	58.92 ± 15.27	0.207^**^	0.508^**^	0.543^**^	0.415^**^	0.554^**^	0.390^**^	0.575^**^	0.425^**^	0.975^**^	0.953^**^	0.781^**^

*FCPS, Family Communication and Problem Solving; USER, Utilizing Social and Economic Resources; MPO, Maintaining a Positive Outlook; AMMA, Ability to Make Meaning of Adversity*;

* *p < 0.05*,

** *p < 0.01*.

### Hierarchical Linear Regression Analysis

Hierarchical linear regression analysis was conducted to identify the relative contribution of the independent variables to psychological resilience (see Table 3). Variables correlated with psychological resilience in the univariate analyses were entered into the model. When the demographic characteristics were controlled in Step 1, SES, which was tested in Step 2, explained an additional 12.8% of variance in psychological resilience. Participants with higher levels of education (β = 0.286, *p* < 0.001) and monthly household income per capita (β = 0.151, *p* = 0.014) reported greater levels of psychological resilience. Family resilience, which was included in Step 3, explained 23.3% of the variance in psychological resilience. Higher scores in the maintenance of a positive outlook (β = 0.325, *p* < 0.001) were indicative of greater levels of psychological resilience. After controlling for the demographics, SES, and family resilience, tangible support (β = −0.135, *p* = 0.037) and positive social interaction support (β = 0.233, *p* = 0.002) emerged as significant predictors of psychological resilience. The addition of social support in Step 4 accounted for 5.5% incremental criterion variance. Furthermore, as shown in Table 3, monthly household income per capita no longer showed predictive utility in the last step (β = 0.096, *p* = 0.058).

**Table 3:** Hierarchical regression analysis of factors influencing psychological resilience.

**Variables**	**Model 1**		**Model 2**		**Model 3**		**Model 4**	
	**β**	**SE**	**β**	**SE**	**β**	**SE**	**β**	**SE**
**Demographic variables**								
Age	−0.104	1.970	−0.063	1.890	−0.090	1.609	−0.061	1.564
Employment status	−0.233*	2.533	−0.178*	2.394	−0.120*	2.304	−0.071	1.989
Marital status	0.194*	2.952	0.221*	2.793	0.181*	2.385	0.134*	2.454
**SES**								
Education level			0.286*	0.987	0.179*	0.858	0.127*	0.836
Monthly household income per capita			0.151*	0.966	0.104*	0.825	0.096	0.790
**Family resilience**								
FCPS					0.171	0.219	0.142	0.212
USER					0.058	0.366	0.043	0.357
MPO					0.325*	0.618	0.269*	0.599
AMMA					−0.011	1.367	−0.030	1.322
**Social support**								
Tangible support							−0.135*	0.289
Informational/emotional support							0.087	0.214
Positive social interaction support							0.233*	0.331
Affectionate support							0.037	0.531
*F*	8.753		14.336		22.982		19.486	
*R* ^2^	0.094		0.221		0.455		0.509	
Adjusted *R*^2^	0.083		0.206		0.435		0.483	
*R*^2^-change	0.094		0.128		0.233		0.055	

*β, Standardized estimate; FCPS, Family Communication and Problem Solving; USER, Utilizing Social and Economic Resources; MPO, Maintaining a Positive Outlook; AMMA, Ability to Make Meaning of Adversity*.

## Discussion

In the present study, maintenance hemodialysis patients reported a low level of psychological resilience (58.92 ± 15.27), which was significantly lower than the normal level in the general population in China (65.46 ± 13.93) (36), but was similar to the level of Chinese cancer patients (57.12 ± 13.56) (37). The treatment process and severe lifestyle changes related to maintenance hemodialysis may reduce the ability of patients to adapt and cope with adversity (38). Since the low psychological resilience of hemodialysis patients is associated with their lower health-promoting behavior and higher level of depression (8), it is highly important for clinical staff to help these patients increase their psychological resilience.

As hypothesized, the results of this study indicated that better SES (higher education level and family income) contributed to a higher level of psychological resilience in maintenance hemodialysis patients, which supports the theory proposed by Wister et al. (20). Maintenance hemodialysis patients with better SES might have more comprehensive understanding of the disease, adopt more effective problem-solving strategies, and have access to more information/health care services (39, 40). Interestingly, the predictive utility of family monthly income was no longer significant when social support was included in the model, which indicates that the family financial level of maintenance hemodialysis patients may affect the psychological resilience through the role of social support. Therefore, it will be more important in future studies to develop more appropriate social support systems for maintenance hemodialysis patients with low SES.

Our results support the hypothesis that family resilience is positively correlated with psychological resilience, indicating that having a family that flexibly responds to changes in a highly challenging environment may lead to positive changes in mental health among hemodialysis patients (41). Maintaining a positive outlook was an independent family resilience factor that influenced psychological resilience in this study, which suggests that maintaining a positive outlook is the most fundamental element of family resilience to foster an individual's psychological resilience (42). Our findings provide some new evidence that a family's shared belief in maintaining a positive outlook is essential to mobilize relational resources to support the positive adaptation of the family and, thus, to guide family members to embrace hope and flexibly respond to hardship when faced with stressful events. Therefore, it is important for families to preserve and nourish their shared beliefs and positive outlook as a way to promote psychological resilience during the process of disease and hemodialysis treatment.

Our findings also support the hypothesis of a positive correlation between social support and psychological resilience. Social interaction support showed a positively predictive effect on psychological resilience, possibly due to the fact that individuals who reported greater support received in positive social interactions would have higher levels of self-efficacy (43) and more resources to cope with stress and the burden of illness (44). Qualitative studies found that positive social interactions gives hemodialysis patients a sense of meaning in life and hope for the future (45), as well as positive emotional experiences and self-worth (46). Thus, nursing interventions focused on promoting positive social interaction support as appropriate and directly or indirectly mobilizing or expanding the social network of the patient may be an effective strategy to improve the psychological resilience of patients. In contrast to our hypothesis, tangible support was negatively predictive of psychological resilience after controlling for other kinds of social support in this study. High tangible support with activities for daily living may threaten self-esteem and the sense of competence and mastery (47). Patients who perceived high tangible support without affectionate supportive communication may view such favors as expressions of obligation rather than a manifestation of love, thereby reducing psychological resilience (48). Therefore, it is important for nurses and caregivers to strike a balance between providing help and maintaining the patient's sense of self-esteem and self-worth, despite the patient's reliance on others for care and support (49).

### Implications for Practice

From a clinical perspective, this study highlights the practical importance of assessing the SES, family resilience, and social support to screen patients with a risk of low psychological resilience, and provides evidence for tailoring family resilience and social support-oriented intervention to improve the psychological resilience of maintenance hemodialysis patients. Specifically, clinical practitioners can conduct family interventions that focus on promoting the shared family belief of a positive outlook toward the disease and treatment to foster individual resilience. Clinical practitioners should also evaluate the sources of social support during treatment and provide appropriate help to guide maintenance hemodialysis patients to seek effective support and enhance their resilience.

### Limitations

There are several limitations of the present study. First, causal relationships cannot be inferred due to the cross-sectional design of this study. Therefore, longitudinal study designs should be used to further explore the complicated dynamic effect of SES, family resilience, and social support on psychological resilience. Secondly, the subjective nature of self-reported questionnaires can lead to reporting bias, especially since psychiatric pathologies and psychological disorders in the participants were not evaluated through specific and structured interviews. However, we initially reviewed the medical records and inquired about the psychiatric history of the patients. Thirdly, family SES was measured only based on the family income and education level in this study. Multiple indices of family SES should be used in future studies. Fourthly, although we adjusted for demographic factors in the analysis, there may be residual confounding factors present, such as the mood of the day and comorbidities. Finally, the generalizability of the results of this study may be limited by convenience sampling. It is necessary to conduct multi-center investigations in future studies.

## Conclusion

Our study showed a low level of psychological resilience among Chinese maintenance hemodialysis patients. The present study demonstrated that SES, represented by education level and family income, is an important predictor of psychological resilience. Maintaining a positive outlook as the most important aspect of family resilience and positive social interactive support positively predicted the patient's level of psychological resilience, while tangible support served as a negative predictor of psychological resilience. Therefore, an approach that focuses on psychological resilience, which patients with lower SES can apply to deal with stress, may reduce the health disparity. In addition, family interventions tailored to maintaining the family's positive outlook or interventions that promote appropriate social support are needed to improve the psychological resilience of maintenance hemodialysis patients.

## Data Availability Statement

The raw data supporting the conclusions of this article will be made available by the authors, without undue reservation.

## Ethics Statement

The studies involving human participants were reviewed and approved by the First Affiliated Hospital of Wenzhou Medical University Ethics Committee (No. 2020198). The patients/participants provided their written informed consent to participate in this study.

## Author Contributions

YQ: data collection, writing-original draft, writing review, and editing. YH, YW, LR, and HJ: data collection, data curation, writing review, and editing. LZ: conceptualization, writing review, and editing. CD: conceptualization, writing-original draft, writing review, and editing. All listed authors meet the authorship criteria and that all authors are in agreement with the content of the manuscript.

## Funding

This work was supported by The National Natural Science Foundation of China (Grant No.72004167) and The Research Projects of the Social Science and Humanity of the Ministry of Education (No. 20YJCZH018).

## Conflict of Interest

The authors declare that the research was conducted in the absence of any commercial or financial relationships that could be construed as a potential conflict of interest.

## Publisher's Note

All claims expressed in this article are solely those of the authors and do not necessarily represent those of their affiliated organizations, or those of the publisher, the editors and the reviewers. Any product that may be evaluated in this article, or claim that may be made by its manufacturer, is not guaranteed or endorsed by the publisher.

## References

[1] Brunner LS Day RA. Brunner and Suddarth's Textbook of Medical-Surgical Nursing. Lippincott: Williams and Wilkins (2018).

[2] Zhao Mh Zhang Lx. China Kidney Disease Network Data Report. Beijing: Science Press (2019).

[3] Abbasi P Mojalli M Kianmehr M Zamani S. Effect of acupressure on constipation in patients undergoing hemodialysis: a randomized double-blind controlled clinical trial. Avicenna J Phytomed. (2019) 9:84–91. 30788281 PMC6369316

[4] Davison SN Jhangri GS. Impact of pain and symptom burden on the health-related quality of life of hemodialysis patients. J Pain Symptom Manage. (2010) 39:477–85. 10.1016/j.jpainsymman.2009.08.008 20303025

[5] Fleishman TT Dreiher J Shvartzman P. Patient-reported outcomes in maintenance hemodialysis: a cross-sectional, multicenter study. Qual Life Res. (2020) 29:2345–54. 10.1007/s11136-020-02508-3 32356278

[6] Lam LW Lee DT Shiu AT. The dynamic process of adherence to a renal therapeutic regimen: perspectives of patients undergoing continuous ambulatory peritoneal dialysis. Int J Nurs Stud. (2014) 51:908–16. 10.1016/j.ijnurstu.2013.10.012 24210362

[7] Mirghaed MT Sepehrian R Rakhshan A Gorji H. Sleep quality in Iranian hemodialysis patients: a systematic review and meta-analysis. Iran J Nurs Midwifery Res. (2019) 24:403–9. 10.4103/ijnmr.IJNMR_184_18 31772913 PMC6875887

[8] Liu YM Chang HJ Wang RH Yang LK Lu KC Hou YC. Role of resilience and social support in alleviating depression in patients receiving maintenance hemodialysis. Ther Clin Risk Manag. (2018) 14:441–51. 10.2147/TCRM.S152273 29535526 PMC5840278

[9] Kim EY Lee YN Chang SO. How do patients on hemodialysis perceive and overcome hemodialysis?: concept development of the resilience of patients on hemodialysis. Nephrol Nurs J. (2019) 46:521–30. 31566347

[10] Pan KC Hung SY Chen CI Lu CY Shih ML Huang CY. Social support as a mediator between sleep disturbances, depressive symptoms, and health-related quality of life in patients undergoing hemodialysis. PLoS One. (2019) 14:e0216045. 10.1371/journal.pone.0216045 31034497 PMC6488079

[11] Connor KM Davidson JR. Development of a new resilience scale: the Connor-Davidson Resilience Scale (CD-RISC). Depress Anxiety. (2003) 18:76–82. 10.1002/da.10113 12964174

[12] Cleary M Jackson D Hungerford CL. Mental health nursing in Australia: resilience as a means of sustaining the specialty. Issues Ment Health Nurs. (2014) 35:33–40. 10.3109/01612840.2013.836261 24350749

[13] García-Martínez P Ballester-Arnal R. Perceived stress in relation to quality of life and resilience in patients with advanced chronic kidney disease undergoing. Hemodialysis. (2021) 18:536. 10.3390/ijerph18020536 33440671 PMC7826655

[14] Noghan N Akaberi A Pournamdarian S Borujerdi E Hejazi SS. Resilience and therapeutic regimen compliance in patients undergoing hemodialysis in hospitals of Hamedan, Iran. Electron Physician. (2018) 10:6853–8. 10.19082/6853 29997771 PMC6033136

[15] Sher L. Sleep, resilience and suicide. Sleep Med. (2020) 66:284–5. 10.1016/j.sleep.2019.08.015 31859035

[16] Dean EE Little L Tomchek S Dunn W. Sensory processing in the general population: adaptability, resiliency, and challenging behavior. Am J Occup Ther. (2018) 72:7201195060p1–8. 10.5014/ajot.2018.019919 29280720

[17] Serafini G Gonda X Pompili M Rihmer Z Amore M Engel-Yeger B. The relationship between sensory processing patterns, alexithymia, traumatic childhood experiences, and quality of life among patients with unipolar and bipolar disorders. Child Abuse Negl. (2016) 62:39–50. 10.1016/j.chiabu.2016.09.013 27792883

[18] Brown ML. Integrative approaches to stress, anxiety, and resilience. Pediatr Ann. (2019) 48:e226–e30. 10.3928/19382359-20190515-05 31185113

[19] De Berardis D Fornaro M Valchera A Cavuto M Perna G Di Nicola M. Eradicating suicide at its roots: preclinical bases and clinical evidence of the efficacy of ketamine in the treatment of suicidal behaviors. Int J Mol Sci. (2018) 19:2888. 10.3390/ijms19102888 30249029 PMC6213585

[20] Wister AV Coatta KL Schuurman N Lear SA Rosin M MacKey D. A lifecourse model of multimorbidity resilience: theoretical and research developments. Int J Aging Hum Dev. (2016) 82:290–313. 10.1177/0091415016641686 27076489

[21] James SA. John Henryism and the health of African-Americans. Cult Med Psychiatry. (1994) 18:163–82. 10.1007/BF01379448 7924399

[22] Cohen S. Social relationships and health. Am Psychol. (2004) 59:676–84. 10.1037/0003-066X.59.8.676 15554821

[23] Chen S. Chinese adolescents' emotional intelligence, perceived social support, and resilience-the impact of school type selection. Front Psychol. (2019) 10:1299. 10.3389/fpsyg.2019.01299 31244719 PMC6579893

[24] Wang A Bai X Lou T Pang J Tang S. Mitigating distress and promoting positive aspects of caring in caregivers of children and adolescents with schizophrenia: mediation effects of resilience, hope, and social support. Int J Ment Health Nurs. (2020) 29:80–91. 10.1111/inm.12651 31917518

[25] Çakir H Küçükakça Çelik G Çirpan R. Correlation between social support and psychological resilience levels in patients undergoing colorectal cancer surgery: a descriptive study. Psychol Health Med. (2021) 26:899–910. 10.1080/13548506.2020.1859561 33347358

[26] Zhang H Zhao Q Cao P Ren G. Resilience and quality of life: exploring the mediator role of social support in patients with breast cancer. Med Sci Monit. (2017) 23:5969–79. 10.12659/MSM.907730 29248937 PMC5744469

[27] Bahremand M Rai A Alikhani M Mohammadi S Shahebrahimi K Janjani P. Relationship between family functioning and mental health considering the mediating role of resiliency in type 2 diabetes mellitus patients. Glob J Health Sci. (2014) 7:254–9. 10.5539/gjhs.v7n3p254 25948449 PMC4802084

[28] Sherbourne CD Stewart AL. The MOS social support survey. Soc Sci Med. (1991) 32:705–14. 10.1016/0277-9536(91)90150-B 2035047

[29] Walsh F. Family resilience: a framework for clinical practice. Fam Process. (2003) 42:1–18. 10.1111/j.1545-5300.2003.00001.x 12698595

[30] Dong C Gao C Zhao H. Reliability and validation of Family Resilience Assessment Scale in the families raising children with chronic disease. J Nurs Sci. (2018) 10:93–7. 10.3870/j.issn.1001-4152.2018.10.093

[31] Kukihara H Yamawaki N Ando M Nishio M Kimura H Tamura Y. The mediating effect of resilience between family functioning and mental well-being in hemodialysis patients in Japan: a cross-sectional design. Health Qual Life Outcomes. (2020) 18:233. 10.1186/s12955-020-01486-x 32680519 PMC7367268

[32] Silva Júnior EGD Eulálio MDC Souto RQ Santos KL Melo RLP Lacerda AR. The capacity for resilience and social support in the urban elderly. Cien Saude Colet. (2019) 24:7–16. 10.1590/1413-81232018241.32722016 30698235

[33] American P. Diagnostic and Statistical Manual of Mental Disorders (DSM-IV-TR), 4th edn. Washington, DC: American Psychiatric Association (2000).

[34] Yu DS Lee DT Woo J. Psychometric testing of the Chinese version of the medical outcomes study social support survey (MOS-SSS-C). Res Nurs Health. (2004) 27:135–43. 10.1002/nur.20008 15042639

[35] Yu NX Zhang J. Factor analysis and psychometric evaluation of the connor-davidson resilience scale (CD-RISC) with Chinese people. Soc Behav Pers. (2007) 35:19–30. 10.2224/sbp.2007.35.1.19

[36] Yu XN Lau JT Mak WW Zhang J Lui WW Zhang J. Factor structure and psychometric properties of the Connor-Davidson Resilience Scale among Chinese adolescents. Compr Psychiatry. (2011) 52:218–24. 10.1016/j.comppsych.2010.05.010 21295229

[37] Zhang F Meng X Ye P. Survey of resilience and its influencing factors among breast cancer patients. Chin J Nurs. (2015) 50:1087–90. 10.3761/j.issn.0254-1769.2015.09.014

[38] Casey JR Hanson CS Winkelmayer WC Craig JC Palmer S Strippoli GF. Patients' perspectives on hemodialysis vascular access: a systematic review of qualitative studies. Am J Kidney Dis. (2014) 64:937–53. 10.1053/j.ajkd.2014.06.024 25115617

[39] Duran S Avci D Esim F. Association between spiritual well-being and resilience among turkish hemodialysis patients. J Relig Health. (2020) 59:3097–109. 10.1007/s10943-020-01000-z 32076996

[40] Karadag E Ugu O Mert H Erunal M. The relationship between psychological resilience and social support levels in hemodialysis patients. J Basic Clin Health Sci. (2019) 3:9–15. 10.30621/jbachs.2019.469

[41] Mackova J Dankulincova Veselska Z Filakovska Bobakova D Madarasova Geckova A van Dijk JP Reijneveld SA. Crisis in the family and positive youth development: the role of family functioning. Int J Environ Res Public Health. (2019) 16:1678. 10.3390/ijerph16101678 31091704 PMC6571796

[42] Walsh F. Applying a family resilience framework in training, practice, and research: mastering the art of the possible. Fam Process. (2016) 55:616–32. 10.1111/famp.12260 27921306

[43] Pham TV Beasley CM Gagliardi JP Koenig HG Stanifer JW. Spirituality, coping, and resilience among rural residents living with chronic kidney disease. J Relig Health. (2020) 59:2951–68. 10.1007/s10943-019-00892-w 31392626

[44] Karadag E Kilic SP Metin O. Relationship between fatigue and social support in hemodialysis patients. Nurs Health Sci. (2013) 15:164–71. 10.1111/nhs.12008 23552015

[45] Raj R Brown B Ahuja K Frandsen M Jose M. Enabling good outcomes in older adults on dialysis: a qualitative study. BMC Nephrol. (2020) 21:28. 10.1186/s12882-020-1695-1 31996167 PMC6988330

[46] West C Stewart L Foster K Usher K. The meaning of resilience to persons living with chronic pain: an interpretive qualitative inquiry. J Clin Nurs. (2012) 21:1284–92. 10.1111/j.1365-2702.2011.04005.x 22404312

[47] Abraído-Lanza AF. Social support and psychological adjustment among Latinas with arthritis: a test of a theoretical model. Ann Behav Med. (2004) 27:162–71. 10.1207/s15324796abm2703_4 15184092 PMC3657203

[48] Guntzviller LM Williamson LD Ratcliff CL. Stress, social support, and mental health among young adult hispanics. Fam Community Health. (2020) 43:82–91. 10.1097/FCH.0000000000000224 31764309

[49] Hoang VL Green T Bonner A. Examining social support, psychological status and health-related quality of life in people receiving haemodialysis. J Renal Care. (2021). 10.1111/jorc.12380. [Epub ahead of print]. 34041850

